# Unilateral proptosis as a neglected case of sphenoid wing meningioma: a case report and healthcare challenges in rural Indonesia

**DOI:** 10.11604/pamj.2023.44.136.38426

**Published:** 2023-03-17

**Authors:** Ivana Beatrice Alberta, Robert Shen, Mardjono Tjahjadi

**Affiliations:** 1 Puskesmas Sumberpitu Primary Health Care, Pasuruan, East Java, Indonesia,; 2 Bunda Pengharapan Hospital, Merauke, South Papua, Indonesia,; 3 Atma Jaya Neuroscience and Cognitive Center (ANCC), School of Medicine and Health Sciences, Atma Jaya Catholic University of Indonesia, North Jakarta, Jakarta, Indonesia,; 4 Department of Physiology, School of Medicine and Health Sciences, Atma Jaya Catholic University of Indonesia, North Jakarta, Jakarta, Indonesia,; 5 Department of Neurosurgery, Mitra Keluarga Hospital Kelapa Gading, North Jakarta, Jakarta, Indonesia

**Keywords:** Cerebral neoplasm, meningioma, orbital neoplasm, rural healthcare, case report

## Abstract

Proptosis, an abnormal protrusion of the eyeball, is a manifestation of a wide variety of pathologies. The complication to be vision- or life-threatening makes early diagnosis is important, especially in rural primary health centers (PHCs) with far-distance referrals to capable hospitals. This case report examines a patient with obvious unilateral proptosis and blurry vision on the right eye for 4 years, with neglect because of prior inadequate diagnosis and explanation before the current complication. Examination shows no light perception, obvious proptosis (30 mm), exodeviation, and ophthalmoplegia. Referral for radiological examination showed a regular extra-axial lesion, well-defined, and broad-based on the right sphenoid wing with hyperostosis. The patient was diagnosed with sphenoid wing meningioma, which complicated into proptosis and blindness. This report aims to explain the current challenges of rural PHCs in Indonesia critically and to emphasize that rural PHCs should overcome the low level of public education, self-awareness of health, and tendency to reject the referral process. Clinicians also have a crucial role in early detection and prompt treatment to reduce further neglected cases.

## Introduction

Proptosis, which refers to “bulging eyes” or exophthalmos, is as an abnormal protrusion (2 mm or greater) of the eyeball from the orbit [1]. Proptosis can be unilateral or bilateral with a wide variety of pathologies. The complication to be vision- or life-threatening makes a critical history taking, physical examination, and supporting investigation is crucial to establish the underlying etiopathology of proptosis including traumatic, infective, inflammatory, endocrine, vascular, and neoplasm causes; although by the age distribution in 40-60 years, inflammatory and neoplasm are accounting for most common etiopathology [1,2]. One of the rare of them are meningiomas, that are uncommon, and only approximately 20% of them are located by the greater sphenoid wing. Herein, we presented a typical case of sphenoid wing meningioma with its progressive classic triad manifestation: proptosis, visual impairment, and ophthalmoparesis; with negligence in screening because of lack of knowledge in primary health center (PHC) doctor, lack of patient's education, and delayed of referral in the first place [3,4].

## Patient and observation

**Patient information:** a 45-year-old female patient came to our rural PHC with a chief complaint of progressive right-eye bulging with blurry right vision that worsened into totally blind. The complaints were initially felt 4 years ago, with recurring headaches, visual disturbances, and painless right-eye bulging; The patient formerly visited another PHC, was given symptomatic therapy, and failed to make a referral to a neurosurgeon and ophthalmologist for further examination and management because of lack of knowledge in PHC doctor causing minimal education regarding her condition and its complication. At that time, the patient said there was a suspicion of neoplasm as a cause, but did not get an explanation for further strategies and complications related to her condition. The patient had no previous history of visual disturbances, progressive weight loss, trauma, use of hormonal contraception, or other systemic diseases and disease-related to inflammation, infection, endocrine disorders, and autoimmune disorders. Hearing loss, anosmia, speech impairment, cognitive impairment, seizure, and paresis were denied. Never had a similar complaint on family history, but the patient's genetic information is unknown. The history of the patient's genetic information is unknown. The patient has an overweight body mass index and sedentary lifestyle, is non-alcoholic, and a non-smoker.

**Figure 1:**
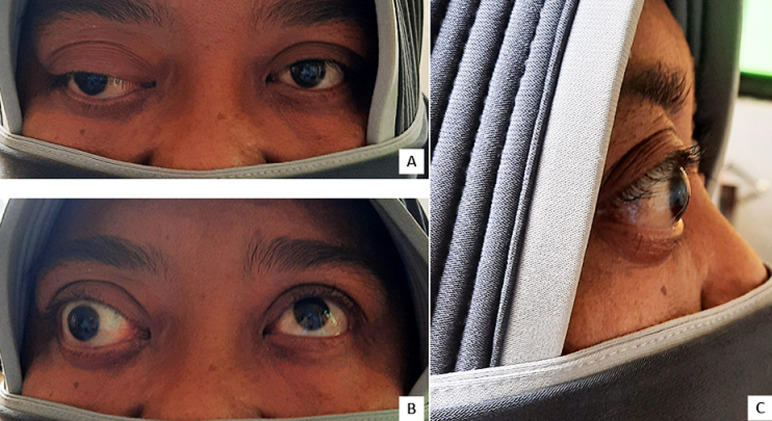
A) inspection in a forward-looking position, showing right-eye proptosis with exodeviation; B) eye movement examination showing right-eye ophthalmoplegia; C) right-eye proptosis (30mm) from a lateral view, measurement using a plastic ruler (not shown in the picture)

**Clinical findings:** on examination, her best-corrected visual acuity was 6/6 in left-eye and there was no light perception (NLP) in right-eye. An obvious proptosis, exodeviation, and ophthalmoplegia, both external and internal, of her right eye was noted (Figure 1), later the measurement of right-eye proptosis using plastic ruler showed 30 mm from lateral orbital rim to the corneal apex compared to the left-eye showed 19 mm within normal limit. Exophthalmometry, tonometry, and funduscopy examination were not performed because the tools are not available.

**Timeline of current episode:** on January 2022, whole events occur in order sequences: admission, anamnesis and data collection, physical examination, referral for radiologic examination using computed tomography (CT) scan and magnetic resonance imaging (MRI), generating working diagnosis, patient education, referral to neurosurgeon.

**Figure 2:**
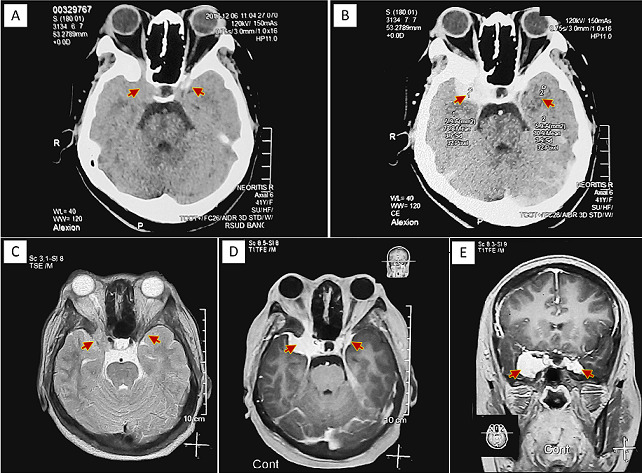
right sphenoid wing meningioma showed (red arrows) by homogenous enhancing solid mass in the right and left suprasellar regions, with hyperostosis; depicted in the axial slice of the; A) non-contrast CT-scan; B) contrast CT-scan; C) non-contrast MRI; D) contrast MRI, and coronal slice of; E) contrast MRI

**Diagnostic assessment:** we explained the patient of the possibility of a slow growing mass just behind the right eye that pushes it outward causing proptosis and compresses the visual and eye movement nerves, causing blindness and impaired eye movement; In addition, we also explained the need for radiological investigation to establish a definite diagnosis and referral to a higher level hospital in a nearby city for further management to prevent complications due to orbital compression and increased intracranial pressure, as well as other neurological disorders that may lead to life-threatening situation. Subsequently, a head CT scan was performed and (Figure 2) showed an extra-axial isodense lesion with 30 Hounsfield Unit (HU) indicating soft tissue lesion, and with contrast injection showed a strong homogenous enhancement (75 HU); the lesion was regular, well-defined, and showed broad-based on the right sphenoid wing. The lesion also appeared to extend toward the left suprasellar region. Marked hyperostosis were visualized on the right sphenoid wing and bilateral temporal bones. Otherwise, within normal limit. Brain MRI also showed homogenous enhancement extra-axial mass lesion upon the right and left suprasellar regions with right sphenoid wing hyperostosis that is regular and well-defined. Thorax X-ray and laboratory testing of complete blood count, electrolytes, liver function, and renal function showed normal results. Unilateral proptosis et causa right sphenoid wing meningioma as the radiological diagnosis has been made.

**Diagnosis:** unilateral proptosis et causa right sphenoid wing meningioma, as the final diagnosis has been made.

**Therapeutic interventions**: there are no therapeutic interventions given in PHC. Further management cannot be carried out at PHC; therefore, the patient is given a one-way vertical referral to a neurosurgeon specialist for consideration of surgery.

**Follow-up and outcome of interventions:** 3-month follow-up after meningioma resection showed the blindness of patient´s right-eye with NLP remained, proptosis in both eyes reduced and showed no deviation, and headache totally resolved.

**Patient perspective:** the patient discovered she could not see in her right eye due to complications from a neoplasm in his brain. The patient thought her eye complaints four years ago could be improved with eye drops. In addition, various traditional and non-medical alternative treatments have also been tried. The patient neglected her complaints because she thought it was God's destiny. In addition, patients also experience problems carrying out health checks and treatment because patients do not have membership in the Indonesia health coverage program due to difficulties in registering due to incomplete data and family files.

**Informed consent:** the authors certify that all written informed consent for medical procedures and patient's medical information study was obtained from the patient to publish this case report and accompanying images. The patient has given her consent for her images and other clinical information to be reported in the journal. All ethical principles for medical research studies established by Sumberpitu Primary Healthcare have been followed, and the ethical clearance committee approved this study.

## Discussion

Retro-orbital tumors are the major etiology (85%) of unilateral proptosis, with meningiomas being the commonest cause [5,6]. However, beside neoplasm, unilateral proptosis also can be caused by orbital cellulitis, developmental or vascular anomalies, inflammatory conditions, and metabolic disease; Hence, the strategy since the case finding in PHC becomes crucial in terms of early screening, confirmative follow-up examinations to rule out differential diagnoses, and proper patient education regarding complications, prognosis, and the importance of referral to the working group of ophthalmologist, neurosurgeon, and radiologist [1]. Learning from our case, inadequate role and indifference of previous investigation can lead to under-diagnosed that cause neglection of proptosis condition and increase the incidence of complications that are detrimental in terms of visual- and neurological-functions and even life-threatening. Besides that, inadequate of education or advice that doctor gave to this patient and the low education level of most rural people had, made her not aware the importance of further workup and treatment regarding her condition; also made this case neglected for almost 4 years. In addition, thoughtful history taking, necessary physical examination, follow-up of progression by photograph and ultrasonography findings also play a role in ruling out unnecessary endocrine studies such as thyroid disease, angiography, and expensive neuro-radiological investigations [7].

The challenges we need to overcome in primary health care as low-middle income countries include: the low level of public education makes it difficult to explain the diagnosis and reasons for supporting investigation, referral and treatment; low levels of self-awareness of health; usually peoples see a doctor when symptoms and complications are severe and irreversible; the gap between rural PHC workers with a dense population causes less time available for examinations to education and therapy per patient; not all doctors in primary health care are aware of cases and complications of proptosis and screening for intracranial neoplasms; there are few or no ophthalmologists and neurosurgeons in Indonesia's periphery, so referrals to other cities are often needed; referrals and relatively expensive surgery tend to be rejected by people who do not have national health insurance and social security card; people still believe in traditional medicine and shamanism to cure various diseases.

Meningiomas are intracranial tumors arising from arachnoid cap cells and are the most common (30%) non-glial central nervous system (CNS) neoplasm in adults among other primary brain tumors [8]. Sphenoid wing meningioma accounts for about 20% of meningiomas cases, with its classic symptoms are proptosis, ophthalmoparesis, and progressive visual acuity impairment, in addition to the general symptoms of meningiomas similar to other extra-axial intracranial tumor manifestations due to compression of CNS [9]. As in our case, extensive hyperostosis is pathognomonic of sphenoid wing meningioma in radiology imaging; a homogeneous hyperdense mass will be seen on CT-scan, which is evenly enhanced by contrast administration. The slow growth of meningiomas makes clinical symptoms not very prominent at first; still, its progressive nature can lead into neurological deficits and ophthalmological complication; whereas they are actually preventable, so that an early diagnosis, referral, and treatment are the key to success in prevent the complications, lower the recurrence, and enhance the patients´ quality of life. Radical resection is still the gold-standard treatment because the meningioma recurrence rate is much higher in partial resection, beside meningiomas are relatively radioresistant to radiotherapy [10].

## Conclusion

Unilateral proptosis has a varied etiopathology but is often caused by retroorbital tumors. The visual and life-threatening complications of proptosis make the role of clinicians in rural PHC very crucial to screening the cause of proptosis and providing adequate education to patients to understand their condition and be willing to be referred for further investigation to make a working diagnosis and determine treatment. Clinicians in rural PHC must understand the health problems in their work-area and find solutions to reduce neglected cases of proptosis or other malignancy diseases manifestations and complications. To support that, it is important to improve the exposure of neurosurgery and knowledge of periocular disease that manifested in ocular symptoms into medical doctor education standard.
